# Pharmaceutical Prospects of Bee Products: Special Focus on Anticancer, Antibacterial, Antiviral, and Antiparasitic Properties

**DOI:** 10.3390/antibiotics10070822

**Published:** 2021-07-06

**Authors:** Firzan Nainu, Ayu Masyita, Muh. Akbar Bahar, Muhammad Raihan, Shajuthi Rahman Prova, Saikat Mitra, Talha Bin Emran, Jesus Simal-Gandara

**Affiliations:** 1 Faculty of Pharmacy, Hasanuddin University, Makassar 90245, Sulawesi Selatan, Indonesia; masyitaa18c@student.unhas.ac.id (A.M.); akbarbahar@unhas.ac.id (M.A.B.); mraihan@unhas.ac.id (M.R.); 2 Department of Pharmacy, Faculty of Pharmacy, University of Dhaka, Dhaka 1000, Bangladesh; shajutirahman19@gmail.com (S.R.P.); saikatmitradu@gmail.com (S.M.); 3 Department of Pharmacy, BGC Trust University Bangladesh, Chittagong 4381, Bangladesh; 4 Nutrition and Bromatology Group, Department of Analytical and Food Chemistry, Faculty of Food Science and Technology, University of Vigo, Ourense Campus, E32004 Ourense, Spain

**Keywords:** honeybee products, anticancer, antibacterial, antiviral, antiparasitic

## Abstract

Bee products have long been used in traditional healing practices to treat many types of disorders, including cancer and microbial-related diseases. Indeed, several chemical compounds found in bee products have been demonstrated to display anticancer, antibacterial, antiviral, and antiparasitic properties. With the improvement of research tools and in view of recent advances related to bee products, this review aims to provide broad yet detailed insight into the pharmaceutical prospects of bee products such as honey, propolis, bee pollen, royal jelly, bee bread, beeswax, and bee venom, in the domain of cancer and infectious disease management. Available literature confirms the efficacy of these bee products in the alleviation of cancer progression, inhibition of bacterial and viral proliferation, and mitigation of parasitic-related symptoms. With such potentials, bioactive components isolated from the bee products can be used as an alternative approach in the long-run effort to improve humans’ health at a personal and community level.

## 1. Introduction

Biologically active bee products are being popular in the current world due to their promising health benefits. The use of honey for nutritional and medicinal purposes dates back nearly 5500 years [1]. Hand collecting honeybees was a significant traditional practice in ancient populations as it was the individual method to get honey, which is still being practiced currently by some people in forest areas [2]. To date, several honeybee products such as honey, bee pollen, royal jelly, propolis, beeswax, bee bread, and bee venom have been identified as prospective sources of compounds with therapeutical potentials in the management of cancer and infections by different types of bacteria, viruses, and parasites [3].

Cancer is one of the major maladies affecting humankind and remains one of the leading causes of death worldwide [4]. Honey, pollen, bee venom, royal jelly, and propolis are commonly used in apitherapy to treat various cancers. Several research studies have reported that bee products are promising candidates for cancer treatment [5,6,7]. Studies have confirmed that honey is an oxidizing agent with pro-apoptotic, anti-proliferative, anti-metastatic, immune-modulatory, and anti-inflammatory properties [6]. Bee venom is a biotoxin or apitoxin synthesized and secreted by a gland in the bee’s abdominal cavity. It appears to be effective in the management of cancer, including the induction of cytotoxicity, necrosis, apoptosis, and proliferation inhibition in various cancer cells, including liver, breast, lung, prostate, and bladder cancer cells [7]. 

Honey is one of the most versatile bee products that possesses a wide range of properties and applications. It is prepared by bees from honeydew and nectar [8]. It contains several bioactive components that differ based on the type of bee and environmental conditions under which it was collected [9]. Honey works as an anti-inflammatory, antioxidant, anti-bacterial agent, etc., and improves the adherence of skin grafts and the facilitates the wound healing process [9]. Propolis, also known as bee glue, is collected by bees from shrubs, tree buds, as well as green plants [3]. It is made up of essential oils, resins, pollen, waxes, and numerous organic compounds, such as amino acids, polyphenols, minerals, flavonoids, ethanol, vitamin E, vitamin B complex, and vitamin A. It works against hive-invading bacteria, viruses, and other pathogenic microorganisms [10]. 

**Figure 1:**
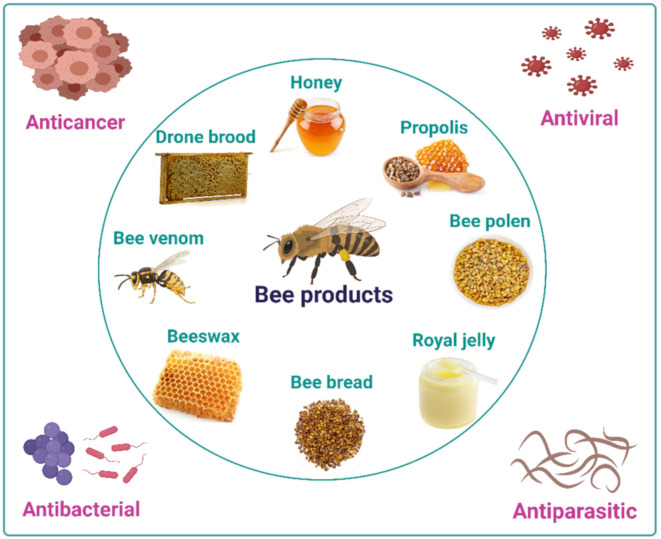
Types of bee products.

Bee products (Figure 1) are also well-recognized due to their antiviral activities, which can outperform standard drugs in some cases. Clinical data suggested that an ointment containing Canadian propolis outperformed placebo or acyclovir in the treatment of genital herpes simplex [3]. Propolis can also inhibit the replication of hepatitis C virus in vitro, inhibit HIV-1 activity by acting on viral integrase, and propolis containing caffeic acid derivatives is potent against herpes simplex 1 and 2 [11,12]. In addition, honey and propolis have also been reported to yield antiviral activities against some human pathogenic viruses such as influenza virus [13], respiratory syncytial virus (RSV) [14], human herpesviruses [15], influenza virus [16,17,18], HIV [19], human T-cell leukemia-lymphoma virus type 1 (HLTV-1) [20], Newcastle disease virus (NDV) [21], RSV [22], poliovirus (PV)-type 1 [23], and dengue virus (DENV) [24]. Other bee products such as bee pollen and bee bread have been shown to be effective as antivirals in an in vitro setting against herpes simplex virus (HSV)-1 and HSV-2 [25] and was moderately active against influenza viruses (strains of H1N1, H3N2, and H5N1) [26]. With their antiviral potentials, bee products such as honey and propolis, have been recently tested in the clinical trials against SARS-CoV-2, an emerging human viral pathogen that has been suggested to cause coronavirus disease (COVID)-19 [27].

In addition to their anticancer, antibacterial, and antiviral activities, bee products have been shown to exert antiprotozoal activity against the nematode model *Caenorhabditis elegans* and the intestinal parasite *Giardia lamblia* [3,28,29]. Studies showed that propolis yielded anti-malarial activities against *Plasmodium vivax*, *P. ovale*, *P. malariae*, and *P. falciparum* [28]. Honey and propolis were also used in ancient times for embalming bodies, and honey in traditional medicine was used for treating wounds and pain relief [8]. Royal jelly, another type of bee product, appears to have significant antimicrobial activity, as reported in several studies [27]. Beeswax, a lipid-based complex organic compound secreted by the younger worker bees in liquid form, forms in solidifies and scales when exposed to air [27,30]. In recent years, the crude extract of beeswax has been reported effective against pathogenic bacteria, viruses, and fungi [31]. Bee pollen is obtained from plants and transferred to hive as pollen loads. Load formation includes hydrating pollen with honey or nectar. In winter, pollen stored in honeycomb cells during winter fermentation undergoes lactic fermentation and produces bee bread. The bacteriostatic and bactericidal properties of bee bread and pollen are also well known [3].

Notwithstanding the immense application of bee products in the medical and pharmaceutical sectors [3], bee products also possess a substantial economic value. Bee products are primarily used in the food and cosmetics industries [8]. Honey is a great sweetener [32], and bee pollen is high in protein, fatty acids, and vitamins [33], suggesting their excellent dietary properties. Royal jelly can improve brain function and general wellbeing [34], while bee propolis and bee venom can be tapped as potential sources of anticancer [35,36] and antiviral [37,38] drug candidates. On the other hand, beeswax has antibiotic and skin softening properties; thus, this particular bee product has been extensively used in the cosmetics industry. This review discusses the importance of bee products in the medical-pharmaceutical fields as well as the potential use and prospective implication of bee products against cancer and different types of microbial pathogens and parasites.

## 2. Types of Bee Products: Description and Components

### 2.1. Honey

**Figure 2:**
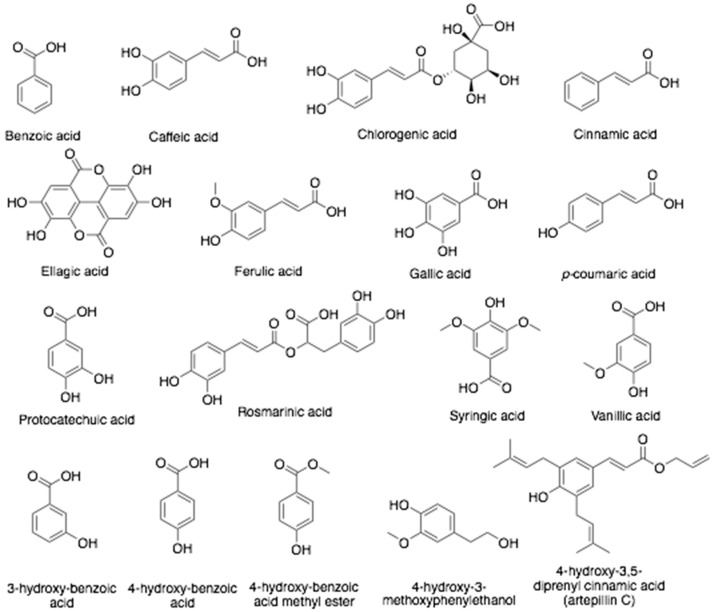
Chemical structures of phenolic acids found in bee products.

**Figure 3:**
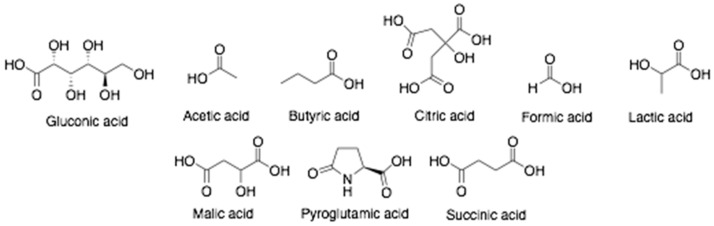
Chemical structures of organic acids found in honey.

**Figure 4:**
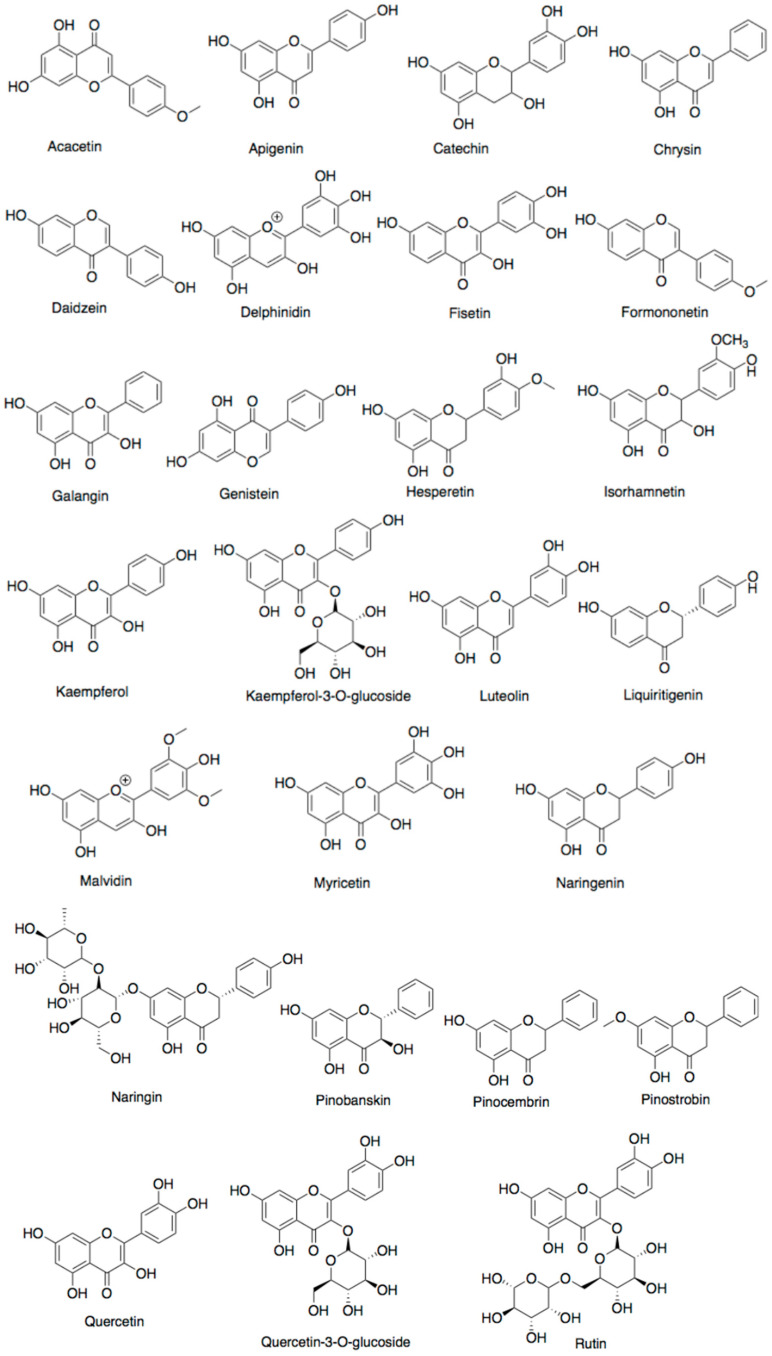
Chemical structures of flavonoids found in bee products.

Honey is a natural food substance generated by honeybees from deposits of plant and floral nectar, which are then coalesced with specific substances of honeybee, processed and stored in honeycombs to ripen [39]. The chemical components of honey depend on several factors including the species of honey-collecting bee, its plant source, climatic conditions, geographical region, and storage condition [40]. Honey consists of enzymes (acid phosphatase, amylase, catalase, diastase, glucose oxidase, invertase, and sucrose diastase) and amino acid monomers (alanine, asparagine, glutamine, glycine, and proline) [1]. Various phenolic acid such as caffeic-, cinnamic-, ferulic-, etc. (Figure 2) and several organic acids, mainly as gluconic and citric acid, followed by acetic acid, formic acid, and others in small amounts (Figure 3) are present in honey. These acids are responsible for the acidic pH of honey ranging from pH 3.4 to 6.1 [41]. Flavonoids (Figure 4) such as quercetin, kaempferol, chrysin, apigenin, hesperetin, galangin, catechin, luteolin, myricetin, and naringenin have also been reported in honey. The total phenolic content (TPC) and the total flavonoid content (TFC) of the honey samples varied from 4.2 ± 0.6 to 1.9 ± 0.1 mg QE/100 g and 31.5 ± 2.1 to 126.6 ± 2.7 mg GAE/100 g, respectively [42].

**Figure 5:**
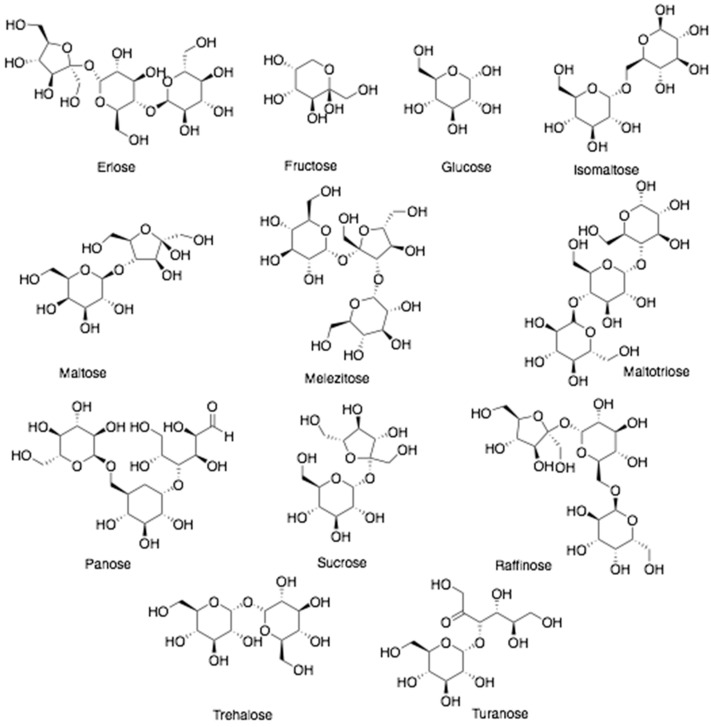
Chemical structures of sugars found in bee products.

Honey is sweet substance predominantly constituted by monosaccharides like fructose (38%) and glucose (31%) as major sugars, with disaccharides (sucrose, gentiobiose, isomaltose, kojibiose, laminaribiose, maltose, maltulose, nigerose, and trehalose) and trisaccharides (centose, erlose, isomaltosylglucose, isomaltotriose, isopanose, 1-ketose, maltotriose, melezitose, panose, and theanderose) in smaller concentrations (Figure 5) [1,43]. Vitamins and minerals are also reported at very low concentrations, about 0.02%, including all of the water-soluble vitamins, and variable minerals such as P, Na, Ca, K, S, Mg, Cl, Si, Rb, V, Zr, Li, Sr [43,44]. In addition, volatile compounds are detected in honey, such as alcohols, aldehydes, benzene and its derivatives, terpene and its derivatives, ketones, pyran, furan, and acid esters [9,45]. The presence of these chemical composition provides better understanding of the beneficial effects of honey such as anticancer, antiallergic, antibacterial, antioxidant, antidiabetic, antiparasitic properties, antiulcer, anti-inflammatory, wound healing, and cardioprotective [3,46,47].

### 2.2. Propolis

Propolis or bee glue is a resinous substance that honeybees produce by mixing their salivary gland excretions with exudate accumulated from different parts of plants, mainly branches, bark, flower buds, leaves, and stems. Propolis comes from two Greek words: pro (defense) and polis (city or community) [48]. The color of propolis is varied, ranging from green to brown and reddish. Propolis possesses a sweet or pleasant odor, and becomes soft and sticky upon heating [49]. Typically, raw propolis consists of resins and balms (50–60%), fatty acids and waxes (30–40%), essential oils (5–10%), and other components (5%) such as enzymes (acid phosphatase, adenosine triphosphatase, glucose-6-phosphatase, and succinic dehydrogenase), vitamins (B1, B2, B6, C, and E), minerals (Mg, Cu, F, Ca, K, Na, Mn, and Zn) [50,51]. Propolis must be purified and dewaxed via solvent extraction to remove inert materials and preserve the phenolic fractions for commercialization [52].

**Figure 6:**
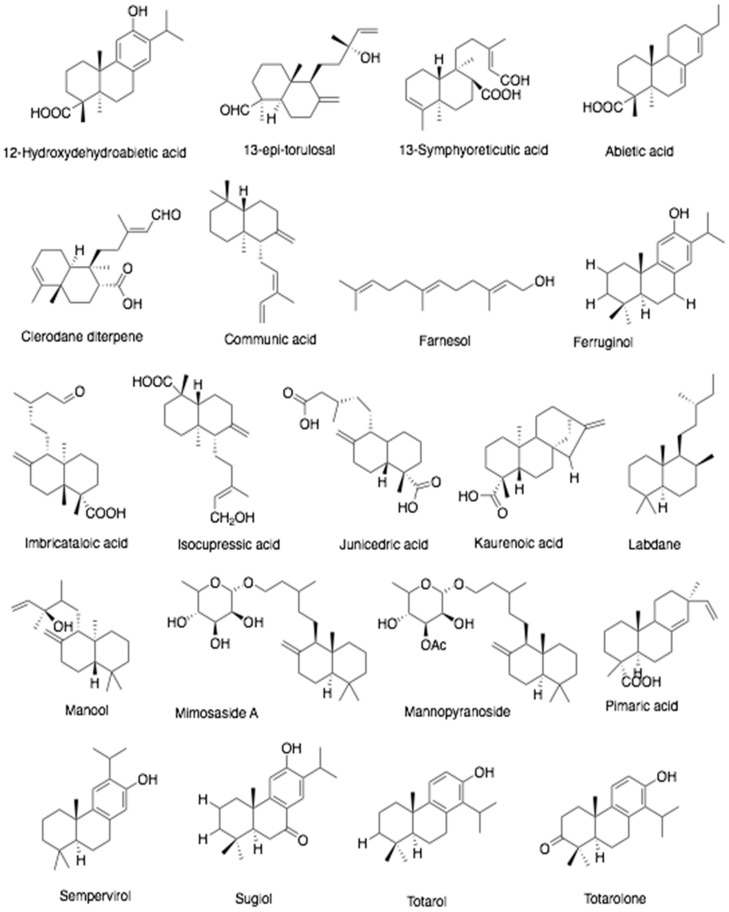
Chemical structures of terpenoids found in propolis.

Several monosaccharides (fructose and glucose) and a disaccharide (sucrose) are found in propolis. It also contains flavonoids (apigenin, chrysin, acacetin, catechin, daidzein, formononetin, naringenin, galangin, kaempferol, luteolin, liquiritigenin, myricetin, pinocembrin, rutin, and quercetin), phenolic acids (caffeic acid, chlorogenic acid, cinnamic acid, gallic acid, 4-hydroxybenzoic acid, 4-hydroxyhydrocinnamic acid, and 4-hydroxybenzoic acid-methyl ester), stilbene derivative (resveratrol), and terpenoids (Figure 6). The TPC and TFC of different samples from different sources such as Brazilian, Chinese, and Australian propolis range from 127–142 mg GAE/g and 33–53 mg QE/g, respectively [53]. Fatty acids such as arachidonic, *cis*-13,16-docosadienoic, *cis*-11,14,17-eicosatrienoic, *cis*-5,8,11,14,17-eicosapentaenoic, eicosadienoic, elaidic, heneicosylic, linoleic, oleic, palmitic, and palmitoleic acid are also present in propolis [49,54]. Propolis and its extracts confers several biological activities, such as antibacterial, anticancer, antifungal, anti-inflammatory, antimycotic, antioxidant, antiulcer, antiviral, cardioprotective, immunomodulatory, neuroprotective, and wound-healing [35,55,56]. 

### 2.3. Bee Pollen

One of the bee products, namely bee pollen, is produced by worker honeybees as the staple food for developing larvae [57,58]. This product results from the mixture of floral nectar, flower pollen, and enzymes with honeybee salivary substances [58]. The chemical compound of bee pollen depends on plant species, bee activities, and weather conditions [59]. The color of bee pollen is diverse, ranging from bright yellow to black and their shapes are also wide ranging: bell-shaped, cylindrical, thorny, or triangular. Bee pollen consist of single grains which are sometimes joined with two or more other grains [60].

Bee pollen has a high carbohydrate content (35–61%), especially of monosaccharides (15–24% fructose and 11–18% glucose), disaccharides (4–9% sucrose) and other sugars such as arabinose, erlose, isomaltose, maltose, melibiose, melezitose, raffinose, rhamnose, ribose, trehalose, and turanose account for about 1% [59,61,62]. Moreover, bee pollen contains protein (14–30%), including essential amino acids (10.4%) such as histidine, isoleucine, leucine, lysine, methionine, phenylalanine, threonine, tryptophan, and valine [60,62,63]. Likewise, bee pollen contains lipids at higher amounts about 1–13% after carbohydrates and proteins. Among fatty acids, the most prevalent saturated fatty acids are myristic, palmitic, stearic acids (4.3–71.5%) while the major unsaturated fatty acids are α-linolenic, linoleic and oleic acids (1.3–53.2%). Arachidonic, behenic, capric, caproic, caprylic, 11-eicosenoic, eicosatrienoic, elaidic, lauric, lignoceric acids also exist in bee pollen [58,64].

Furthermore, bee pollen was reported to contain flavonoids, phenolic acids, and tannins [58]. The total phenolic content (TPC) and total flavonoid content (TFC) of bee pollen of distinct countries are 0.50–213 mg GAE/g and 1.00–5.50 mg QE/g, respectively [33]. The main flavonoids of bee pollen are present in about 1.4% including isorhamnetin, kaempferol, quercetin and its 3-O-glucosides, followed by apigenin, catechin, epicatechin, hesperetin, luteolin, naringenin [60,65]. On the other hand, the glucosides of anthocyanins, delphinidin, malvidin, and petunidin, were identified in bee pollen from Spain [66]. Bee pollen contains the following phenolic acids: caffeic, chlorogenic, ferulic, gallic, *p*-coumaric, *p*-hydroxybenzoic, protocatechuic, rosmarinic, syringic, and vanillic acid [58]. Bee pollen consists of vitamins, both water-soluble 0.6%, such as vitamin B1, B2, B3, B5, B7, B6, B8, B9, C, and vitamin P; and fat soluble 0.1% such as provitamin A (β-carotene), vitamin E and D [67]. Minerals also belong to valuable substances in bee pollen, including macronutrients (Ca, K, Mg, Na, P) and micronutrients (Fe, Cu, Cr, Mn, Se, Si, Zn) [68]. Accordingly, bee pollen was reported as a valuable dietary supplement with therapeutic properties, including antibacterial, antioxidant, anti-atherosclerotic, anticancer, antiallergenic, anti-fungicidal, chemo-preventive, hepatoprotective, and immunomodulatory activities [69,70,71].

### 2.4. Royal Jelly

Royal jelly (bee’s milk) is a viscous whitish to yellow jelly-like substance secreted by the mandibular and hypopharyngeal glands of worker honeybees [72,73]. It is slightly soluble in water with a strong smell and sour or sweet flavor and a pH of 3.1–3.9 [74]. Royal jelly plays an important role in honeybee larvae diet. It is fed exclusively to young larvae of workers and drones in their maturation process, and is provided to queen honeybees during their entire life cycle [75]. Generally, royal jelly contains water (50–70%), carbohydrates (30%), proteins (27–41%), and lipids (3–19%) [48,76]. The major sugars present in royal jelly include fructose and glucose. Moreover, sucrose and other oligosaccharides like erlose, gentobiose, isomaltose, maltose, melezitose, raffinose, and trehalose are present in very small concentrations [76,77]. A unique group of nine soluble major royal jelly proteins (MRJPs 1–9) functions as the specific factors responsible for development of queen honeybees. The peptides of royal jelly including apisimin, jelleines, and royalisin, have been shown to yield antibacterial activity [3].

**Figure 7:**
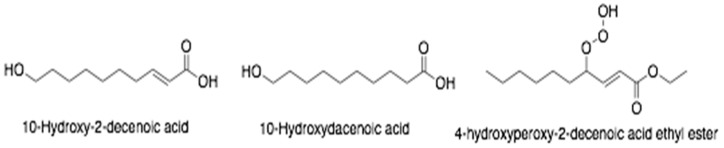
The main fatty acids of royal jelly.

The lipid composition is reported as fatty acids (80–85%), waxes (5–6%), steroids (3–4%) and phospholipids (0.4–0.8%). Royal jelly fatty acids (Figure 7) are usually either uncommon short chain hydroxy and dicarboxylic acids (8–12 carbon atoms) such as 10-hydroxy-*trans*-2-decenoic acid (10-HDA) and 10-hydroxydecanoic acid (HDAA), *trans*-2-decenoic acid, 24-methylenecholesterol, 4-hydroxyperoxy-2-decenoic acid ethyl ester, and sebacic acid, 3-hydroxydecanoic, 9-hydroxy-2-decenoic, 8-hydroxyoctanoic, and 9-hydroxydecanoic acid. Among them, 10-HDA and 10-HDAA are specific compounds of royal jelly [78,79,80].

Royal jelly consists of flavanones (pinobaskin, pinocembrin, hesperidin, naringin, and naringenin), flavones (acacetin, apigenin, chrysin, and luteolin), flavonols (fisetin, galangin, isorhamnetin, kaempferol, quercetin, and rutin), phenolic acids (caffeic acid, gallic acid, 4-hydroxy-3-methoxyphenylethanol, 4-hydroxybenzoic acid-methyl ester, 4-hydroxybenzoic acid, 4-hydroxyhydrocinnamic acid, octanoic acids, 2-hexenedioic acid and its esters, dodecanoic acid and its ester, 1,2-benzenedicarboxylic acid, and benzoic acid) and other phenolic compounds such as hydroquinone, methyl salicylate, 2-methoxy-p-cresol, 2-methoxyphenol, and pyrocatechol [48,76]. The TPC and TFC range from 3 to 9 mg GAE/g and 0.1 to 0.5 mg QE/g royal jelly, respectively [74]. In addition, royal jelly contains hormones (prolactin, testosterone, estradiol, and progesterone), minerals (Cu, Fe, K, Mg, and Zn), and vitamins (B1, B2, B3, B5, B6, B9 and provitamin A) [79].

A variety of chemical components found in royal jelly exhibit several pharmacological properties, including antiaging, antiallergic, antibacterial, anticancer, antidiabetic, anti-inflammatory, hypoglycemic, hypotensive, hepatoprotective, immunomodulatory, and neuroprotective effects [81,82,83].

### 2.5. Bee Bread

Bee bread (ambrosia) is a mixture of honey, pollen, and honeybee salivary secretion, which is then stored in the beehive and enclosed with honey and wax [84]. Bee bread is also called “fermented bee pollen” due to during the preservation, as the content is subjected to lactic fermentation in the beehive environment [85]. Therefore, the color of bee bread is caramel with a pungent flavor. Bee bread is the main food for larvae and the young worker bees that produce royal jelly [84].

Bee bread contains proteins, enzymes (saccharase, amylase, phosphatase, pepsin, trypsin and papain), minerals (Ca, Mg, P, K, Fe, Zn, and Mn), and vitamins (B-complex, C, D, and E, mainly vitamin P and β-carotene). Vitamin K, also present in bee bread, is not found in fresh bee pollen. Most kinds of bee bread contain 24–34% of carbohydrates. Flavonoids (kaempferol, isorhamnetin, apigenin, chrysin, naringenin, and quercetin) and phenolic acids (*p*-coumaric, caffeic, ferulic and gallic acids) also show high concentrations [86]. The TPC and TFC of bee bread samples varies from 8.26 ± 0.299 to 43.42 ± 0.779 mg GAE/g and 1.81 ± 0.040 to 4.44 ± 0.125 mg QE/g, respectively [87]. 

**Figure 8:**
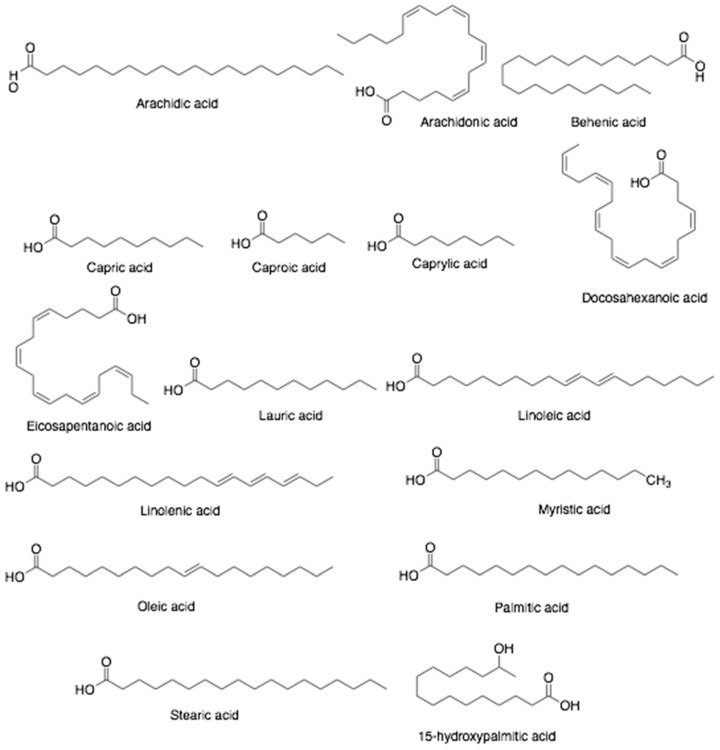
Chemical structures of fatty acids found in bee products, including bee bread.

Fatty acids (Figure 8) such as arachidic acid, arachidonic acid, docosahexaenoic acid, eicosapentaenoic acid, linoleic acid, myristic acid, oleic acid, palmitic acid, and α-linolenic acid, (9*Z*,12*Z*)-octadeca-9,12-dienoic, (9*Z*,12*Z*,15*Z*)-octadeca-9,12,15-trienoic, (*Z*)-octadec-9-enoic, (*Z*)-icos-11-enoic, hexadecanoic and octadecanoic acids were also detected in bee bread [88]. The content of lactic acid in bee bread is higher than 3%. The pH value of bee bread is 4.2, mainly due to the formation of lactic acid [86]. Bee bread was reported to have biological activities including antioxidant, antibacterial, antitumor, antihypertensive, neuroprotective, and antiseptic [52,89,90,91].

### 2.6. Beeswax

**Figure 9:**
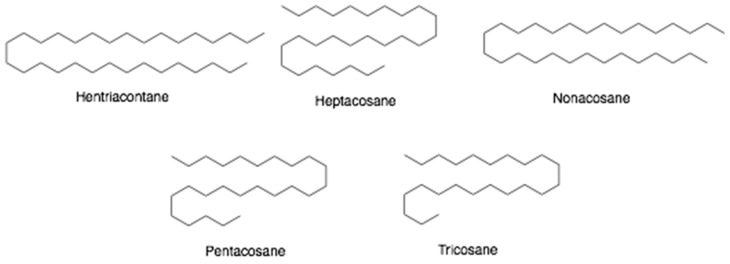
Chemical structures of hydrocarbons found in beeswax.

Beeswax is a liquid substance secreted by the wax glands of younger worker honeybees that is used in the construction of the honeycombs. The color of beeswax turns from white to yellowish-brown after contact with honey and bee pollen. It dissolves completely in chloroform and partially in boiling alcohol [31]. Generally, beeswax consists of more than 300 components, including hydrocarbons (12–16%, Figure 9), mainly heptacosane, nonacosane, hentriacontane, pentacosane and tricosane; free fatty acids (12–14%) such as 15-hydroxypalmitic acid, oleic acid, and palmitic acid; linear wax monoesters and hydroxy monoesters (35–45%), complex wax esters (15–27%) containing 15-hydroxypalmitic acid or diols. Vitamins (A, B1, B4, B6 and P) and minerals (Ca, Cu, Fe, K, Mn, Na, P, and Zn) are also present in beeswax. Beeswax is used in the food, pharmaceutical, and cosmetic industries as an additive [31,52]. In addition, beeswax exhibits antimicrobial activities against *Staphylococcus aureus*, *Salmonella enterica*, *Candida albicans* and *Aspergillus niger* [31].

### 2.7. Bee Venom

**Figure 10:**
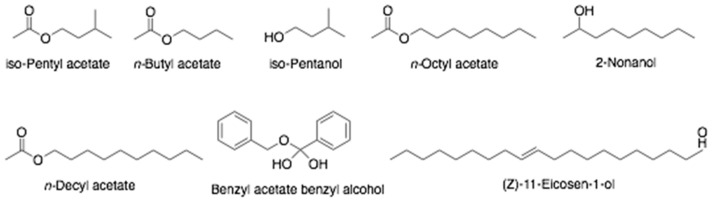
Chemical structures of volatile compounds found in bee venom.

Bee venom or apitoxin is a clear liquid secreted by the venom gland of honeybee located in the abdominal cavity. It is injected into victims by a stringer, causing an immunological response, mainly inflammation [3]. Bee venom is soluble in water with a pH 5–5.5. Bee venom is highly hydrophilic due to the fact more than 80% of BV is water. Bee venom constituents include enzymes, predominantly two allergenic peptides: phospholipase A2 and hyaluronidase (Api m2), followed by icarapin, two serine proteases: Api SI and Api SII, and acid phosphatase or Api m3 [92,93]. Minerals such as Ca, Mg, and P are present in measurable concentrations. Volatile compounds are detected in bee venom such as isopentyl acetate, *n*-butyl acetate, isopentanol, *n*-hexyl acetate, *n*-octyl acetate, 2-nonanol, *n*-decyl acetate, benzyl acetate, benzyl alcohol, and (*Z*)-11-eicosen-1-ol (Figure 10) [36].

**Figure 11:**
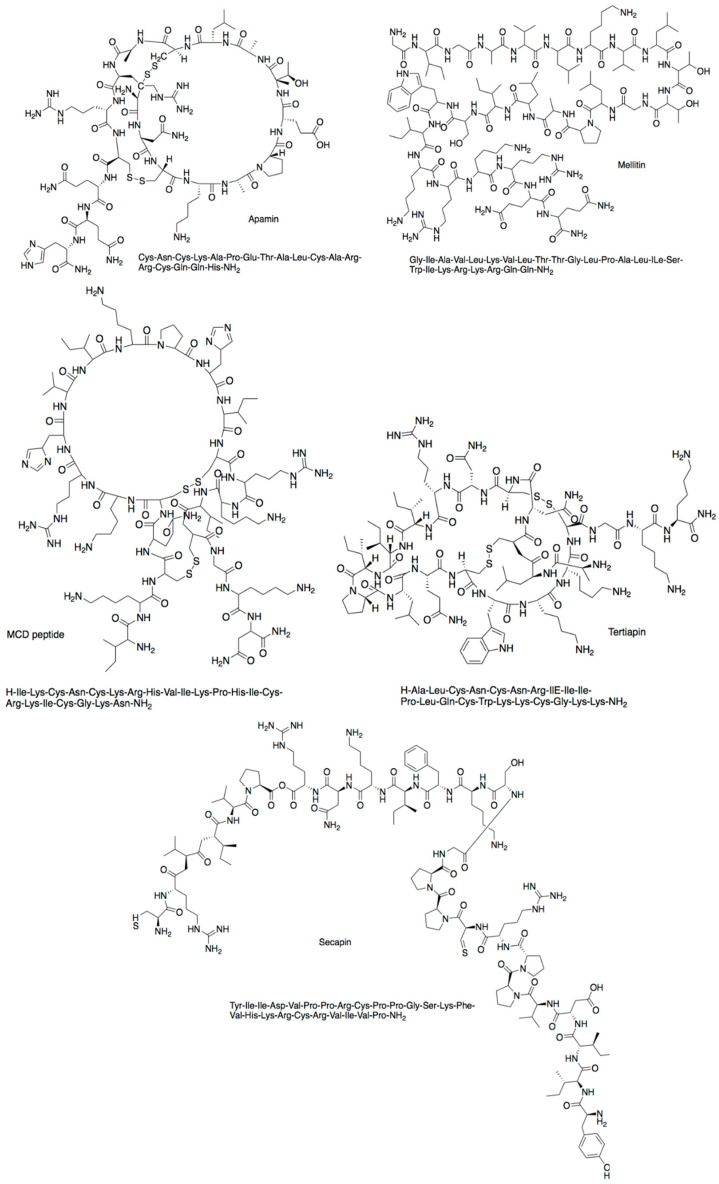
Chemical structures of peptides found in bee venom.

Furthermore, bee venom is composed of a very complex mixture that contains more than 18 active components, including peptides (Figure 11), proteins, enzymes, sugars, amines, phospholipids, pheromones, and volatile compounds [94]. The major amphipathic polycationic peptides, mellitin and apamin, which are a unique component of bee venom. Other peptides such as mast-cell degranulating (MCD) peptide, adolapin, tertiapin, secapin, and cardiopep also present in bee venom [94,95].

Bee venom was investigated as a potential Parkinson’s disease therapy and rheumatoid arthritis, anti-inflammatory, antioxidant, antibacterial, anticancer, antimutagenic, antinociceptive, radioprotective, analgesic, immunomodulatory, anti-apoptotic or anti-secretory activity [7,96,97].

### 2.8. Drone Brood

Drone brood or apilarnil is a little-known bee product acquired by the collection of drone larvae from drone cells (3–11 days after hatching) [98]. Drone brood is a milky, sweet substance with a slightly acidic taste. The odor of drone brood is similar to that of royal jelly. Drone brood is a tenacious substance of creamy consistency with a yellowish gray color [98]. 

Drone brood consists of 9–12% protein, including amino acids, mainly 6.5% glutamic acid, 2.1% alanine, 3.6% aspartic acid, 3.6% leucine, 2.9% lysine, 3.4% proline, 2.3% valine, and followed by histidine, methionine, phenylalanine, and tryptophan. Drone brood contains carbohydrate (6–10%), lipid 5–8%, ash 2%, dry matter (approximately 25–35%), hormones (female sex hormones: estradiol, prolactin, progesterone; and male sex hormones: testosterone), vitamins (B1–B6, A, D, and E), and minerals (Mg, Ca, P, Na, Zn, Mn, Fe, Cu, K, and Se). The biological activity of drone brood includes antioxidant, antiatherosclerotic, androgenic and adaptogenic effects [99].

## 3. Anticancer Properties of Bee Products 

There is currently growing interest in bee products particularly in terms of their potential anticancer activities. It has been previously reported that some bee products can interfere with the development of cancer cells. In this review, we highlight several studies regarding the most recent anticancer activities of bee products (summarized in Table 1). In addition, we also discuss the potency of each presented bee product and the possible mechanisms by which the products or their constituents act in inhibiting the cancer cell growth.

As one of the most utilized bee products, honey has been an undoubtedly an important bee product not only because of its nutritional values but also its medicinal properties. In terms of anticancer activities, honey exerts cytotoxicity against several cancer cell lines. For instance, in an MTT assay, honey samples obtained from Morocco decreased the cell viability of human colorectal cancer (HCT-1) cell cultures [100]. Further investigation to identify the constituents in the active honey samples revealed that phenolic compounds such as rosmarinic acid, tannic acid, caffeic acid, coumaric acid, gallic acid, ferulic acid, syringic acid, catechin, and pyrogallol were present [100]. Meanwhile, manuka honey is reported to actively inhibit the proliferation of MCF-7 at various concentrations [101,102,103,104]. Acacia honey also exhibited anticancer activity against MCF-7 at a concentration of 5.5% *v*/*v* [105]. Beside HCT-1 and MCF-7, honey (0.5 to 1 mg/mL) was also reported to inhibit the growth of PC-3, a prostate cancer cell model [106]. 

These anticancer activities are suggested to be influenced by the substances that are present in honey and hence, correlate to its mechanisms in inhibiting the growth of cancer cells [107]. In general, honey consists of inverted sugar like glucose and fructose at a relatively high concentration but some compounds such as flavonoids, polyphenols, amino acids, carotenoids, vitamins and minerals may also be found [107,108]. Other phytochemicals such as simple polyphenols and flavonoids (chrysin, apigenin, caffeic acid, chrysin, galangin, kaemfereol, pinocembrin, pinobanksin and quercetin) can also be found [109,110]. In the evaluation of the anticancer activity of chestnut honey, a quinoline alkaloid was shown to be responsible for the apoptosis mechanism against castration-resistant prostate cancer (CRPC) cells [111]. Other mechanisms by which honey and its constituents interfere with the development of cancer cells are prevention of cellular damage by free radicals by the antioxidant constituents in honey, induction of apoptosis via cellular signalling pathways and immunomodulation activity, and estrogenic effects [102,107,111,112].

The antiproliferative potencies of propolis have also been studied extensively in recent years. Unlike honey, propolis is not usually tested in a form of a raw product, but rather, it is extracted using methanol, ethanol or other organic solvents before the pharmacological activities are evaluated because of its resinous consistency. Cytotoxic tests against A549 cells, a model of human lung cancer cell, revealed that propolis extract obtained from Turkey indicated inhibition of cell growth [113]. The ethyl acetate fraction of propolis from Saudi Arabia is reported to inhibit Jurkat cells (a T-lymphocyte leukemia model), as well as human liver carcinoma cells (HEP-62) and squamous carcinoma (SW-756) cell lines [114]. Similarly, propolis from Lebanon was also reported to suppress the growth of Jurkat cells [115]. Interestingly, when tested in other carcinoma cell models such as U251 (glioblastoma) and MDA-MB-231 (breast adenocarcinoma), the hexane fraction is the only fraction to show inhibition against these cell models compared to the aqueous and dichloromethane fraction [115]. These results suggest that less polar substances in the propolis may be responsible for the anticancer activities. 

Simple polyphenol compounds such as caffeic acid, chrysin, *p*-coumaric acid, galangin, ferulic acid, and pinocembrin are among the most reported phytochemicals to be found in propolis. These compounds have also been suggested to play significant roles in the suppression of cancer cell growth. Czyewska compared the anticancer activity of extracted propolis to the mixture of polyphenols containing chrysin, galangin and *p*-coumaric acid using CAL-27 cells, a human tongue squamous cancer model. Although the results also showed that the mixture of polyphenolic compounds exhibited higher cytotoxicity than the propolis extract [116], it is important to note that the mixture was tested at higher concentration of polyphenols instead of mimicking the relative concentration each substance found in the tested propolis. One possible mechanism in which propolis may interfere with the development of cancer is by the enhancement of the immune system. As an example, propolis samples from northern Morocco which are reported to be cytotoxic against MCF-7, HCT and THP-1 are shown to enhance production of interleukin-10 (IL-10) and decrease TNF-α and IL-6 production [117], suggesting an immunomodulatory activity of this propolis as a possible mechanism to combat the tested cancer cells. The other mechanisms underlying the anticancer activities of propolis are predicted to be related to its ability to interact with microtubules and induction of tubulin depolymerisation [114], activation of apoptosis via caspase-3, -8 and -9 [116], and reduction of proline in cancer cells via proline dehydrogenase/proline oxidase activity [118].

Bee pollen is another bee product that has been examined for its anticancer properties. Compared to other bee products, bee pollen seems to yield a relatively weaker anticancer potency. In an in vitro assay of anticancer activities using mouse B16 melanoma cells, up to 100 μg/mL of bee pollen was not able to reduce the cultured cell viability [119]. However, it inhibits intracellular tyrosinase (TYR) and interfere with the expression of mRNA corresponding to TYR and tyrosinase receptor, TRP-1 and TRP-2 [119]. Bee pollens collected from different places in South Korea were tested against human prostate adenocarcinoma (PC-3), human lung carcinoma (NCI-H727), human lung carcinoma (A549), MCF-7, and AGS, resulting in IC_50_ values between 0.9 to >25 mg/mL [120]. Stronger anticancer properties were shown by enzymatically cleaved bee pollen proteins, also known as the hydrolysates. It was reported that the hydrolysate peptides lower than 65 kDa in molecular weight were able to inhibit ChaGo-K1 cells, a human bronchogenic carcinoma model, at an IC_50_ of 1.37 μg/mL [121]. From the above data, it is known that higher concentrations of bee pollen are required to inhibit certain cancer cell lines. However, it should also be seen as a sign that bee pollen may be less toxic to normal cells although we did not describe its toxicity profiles in this review.

Bee venoms have also been reported to exhibit anticancer properties [122,123]. One of the most notable components in bee venom is melittin, a major protein substituent found in most venoms of bee species under the *Apis* genus. Melittin from *Apis florea* and *Apis mellifera* have been shown to exhibit a relatively strong anticancer activity (IC_50_ = 3.38 and 4.97 μg/mL, respectively) when challenged against A375 (human malignant melanoma), comparable to that of doxorubicin [124]. A cytotoxicity examination of melittin against HeLa, WiDr and Vero cell lines was also reported, showing anticancer activities with IC_50_ values of 2.54, 2.68 and 3.53 μg/mL, respectively [125]. Melittin also exerts cytotoxic activity against MDA-MDB-231, a human breast cancer cell line, with an IC_50_ of 6.25 µg/mL [126]. At a concentration of 0.5 µg/mL, melittin is able to reduce the viability of cultured AGS cells, a gastric cancer model [127]. The anticancer mechanism of melittin is possibly related to its ability to activate the apoptotic pathway via cytochrome-c discharge and therefore activates caspase-9 which leads to the activation of caspase-3 [124]. In relation to this, further investigation was carried out which indicated that melittin prevents the invasion and migration of melanoma cells in a metastatic cell model, mainly though interference with F-actin reorganization and epidermal growth factor receptor (EGFR) activation [124]. Although it is encouraging that melittin seems to be a promising anticancer agent, there is a growing concern that this protein may also be active against normal cells. Besides, bee venom in general is also highlighted for its adverse cytolytic effects. Therefore, measures to avoid or minimize the disadvantages of bee venom administration in cancer therapy have been attempted. Some of the solutions to this problem are the application of specialized drug delivery systems, i.e., nanoparticles, to carry the toxin protein [128,129], and conjugation of the toxin to specific cancer-targeting biomolecules [7,130,131].

The anticancer potential of other bee products such as royal jelly and bee bread was also reported. Royal jelly’s effect on mammary cancer has been examined using 4T1 cells inoculated in mice. The results revealed that the tumor weight was significantly reduced and further evaluation of the mechanisms revealed changes in interleukin (IL)-2, IL-10 and interferon (INF)-α concentrations in mice plasma [132]. In a recent review regarding the anticancer activity of royal jelly, it was highlighted that the main compound in royal jelly that is thought to be responsible for its anticancer activity is called 10-hydroxydecenoic acid (10-HDA), since it is exclusively found in royal jelly (among the other bee products) at relatively high concentration [133]. However, in another study, it was reported that royal jelly or 10-HDA alone were not effective in inhibiting the growth of human colorectal carcinoma (Caco-2) cells but a mixture of royal jelly and human IFN-α3N at a ratio of 2:1 significantly reduced the cell viability [134]. Miyata et al. expanded the research further to test the anticancer potency of royal jelly in a randomized double-blinded clinical trial. Although the anticancer activity of royal jelly was found to be insignificant, there was a reduction on the adverse events frequencies among patients receiving royal jelly as adjuvant for tyrosine kinase inhibitors [135,136]. In contrast, Osama et al. reported that although a certain potency of royal jelly in protecting the renal functions of patients is observed, it was found to be insignificant in anticancer therapy of cisplatin [137]. Apart from that, the investigation on the mechanisms in the activity of royal jelly as anticancer revealed that it may enhance production of cytokine from mononuclear cells to suppress the growth of U937, a leukemia cell model [138]. Meanwhile, bee bread, a bee product that is closely related to royal jelly, was also shown to have antiproliferative activities against Caco-2 and PC-3 cell lines [139]. Bee bread has also been tested against MCF-7, HeLa, HepG-2 and non-small cell lung cancer (NCI-H460), although the potency was relatively low to moderate (GI_25_ > 400 to 68 μg/mL) [89]. It consists mainly of polyunsaturated and monounsaturated fatty acids [139], but the substances that are thought to be responsible for the anticancer potency are its flavonoids and polyphenolic constituents including isorhamnetin-*O*-glycoside, quercetin-*O*-glycoside, herbacetin glycosides, kaempferol, and myricetin [89].

In general, the anticancer activities of bee products presented in this review reveal that bee products are potential sources of anticancer agents with a wide range of cytotoxic mechanisms. We are aware that anticancer activities of the bee products were mostly assessed using in vitro MTT assays. Hence, a detailed evaluation on these products against cancer-bearing animal models is required to obtain a deeper insight on the influence of different factors on the potencies of these natural products. Additionally, the toxicity profiles of each bee products against normal cells should be evaluated since many anticancer agents are not only toxic to cancer cells but also to normal tissues.

## 4. Bee Products as Prospective Sources of Antibacterial and Antiviral Agents

Bacterial and viral infections are two of the top causes of deaths worldwide. An increasing number of reports describing the development of bacterial and viral resistance, including in the form of polymicrobial infections, against currently available antibiotics and antivirals has urged the use of alternative products with potential activities against those two types of pathogens [141,142,143,144]. One of the commodities equipped with such activities are bee products [3,145,146,147,148,149]. Bee products such as honey, propolis, bee pollen, royal jelly, beebread, and bee venom have been broadly used in the traditional healing practices, including in the management of infectious diseases [49,147,150,151]. A selected list of bee products with antibacterial properties can be seen in Table 2. With their enormous medical and pharmaceutical potentials, bee products shall be considered as one of best prospective sources to discover novel antibacterial and antiviral drugs.

Honey is comprised of more than 150 different substances, including nutrients such as carbohydrates, proteins, vitamins, minerals, water, and different types of polyphenolic compounds [149,150]. Geographical setting and climate condition have been suggested to play a decisive role in determining the composition and concentration of active compounds in the nectar [151], thus the quality and, subsequently, the antimicrobial and antiviral activities of the blossom honey can vary from one to another.

Honey exerts broad spectrum antimicrobial efficacy against different types of pathogenic bacteria [152] and viruses [153]. The antibacterial activities of honey are influenced by numerous physical and chemical properties such as high sugar content (high osmolality), low pH, glucose oxidase activation that leads to hydrogen peroxide production, and in addition to that, the biological action of chemical compounds present in honey such as bacteriocins, bee defensin, methylglyoxal, 3-phenyllactic acid (PLA), and the so-called Major Royal Jelly Proteins (MRJPs) [154]. Honey has been shown to yield exceptional antibacterial activities against both Gram-positive (including methicillin-resistant *S. aureus* (MRSA)), and Gram-negative bacteria, which are frequently linked to skin infections [155]. Manuka honey, a type of honey derived from *Leptospermum scoparium*, has been reported to have a strong antibacterial activity against *S. aureus*, *S. epidermidis*, *Enterobacter aerogenes*, *Salmonella enterica* serovar Typhimurium, *Klebsiella pneumoniae*, and *Escherichia coli* [156].

Honey has been reported to yield biological effects not only against bacterial pathogens but also against human pathogenic viruses, including the latest threat of SARS-CoV-2 [157]. Overall reports indicate that honey is a prospective sources of antiviral compounds with excellent in vitro efficacy against varicella zoster virus (VZV) [158] and rubella virus [159]. Honey, either in a single use or in a combination with other products, has also been reported to demonstrate antiviral activity against influenza virus [13], herpes simplex virus (HSV)-1 [160], and respiratory syncytial virus (RSV) [14]. In addition, honey can improve the life of patients infected with human immunodeficiency virus (HIV) by promoting the proliferation of lymphocytes and by maintaining the hematological and biochemical parameters at optimal conditions [160,161].

The antibacterial activity of other types of bee products such as propolis, bee pollen, royal jelly, bee bread, and bee venom have also been reported [3,145,148,162]. Propolis exerts its antibacterial potential using two distinct mechanisms: either by promoting the activation of host immune responses (indirect action) or via direct interaction of its component(s) with certain parts of bacteria, for example by disruption of cell wall synthesis and alteration of membrane potential [148,163]. Research carried out by a Brazilian group demonstrated the antibacterial activity of propolis against MRSA [164], most likely due to the presence of artepillin C. Separate studies by Japanese and Chilean groups confirmed the effectiveness of propolis against *Porphyromonas gingivalis* [165] and *Streptococcus mutans* [166], respectively, suggesting the potential use of propolis in the management of periodontal diseases. In addition, the high content of kaempferide, artepillin C, drupanin and *p*-coumaric acid present in the ethanolic extract of propolis has been shown to positively correlate with its excellent antioxidant and antimicrobial activity against diverse types of pathogenic bacteria, including *S. aureus*, *S. saprophyticus*, *Listeria monocytogenes,* and *E. faecalis* [167]. In addition to its antibacterial effect, propolis has also been reported to exert antiviral activity against many human pathogenic viruses, including human herpesviruses [15], influenza virus [16,17,18], HIV [19], human T-cell leukemia-lymphoma virus type 1 (HLTV-1) [20], Newcastle disease virus (NDV) [21], RSV [22], poliovirus (PV)-type 1 [23], and dengue virus (DENV) [24]. Recently, flavonoids of propolis and honey such as rutin, naringin, and quercetin, have been suggested as candidates for potential adjuvant treatment against SARS-CoV-2 [168]. 

Bee-collected pollen, simply called bee pollen, and bee bread are two bee products commonly known for their dietary value [145]. Based on the published literature, bee pollen and bee bread demonstrate good antimicrobial activities against several human bacterial and viral pathogens [145]. However, like honey and propolis, the antimicrobial activities of bee pollen and bee bread are varied, and largely dependent on the geographical source of the collected samples and the solvents used in the extraction process [145]. Chilean bee pollen extracts inhibited the growth of *Streptococcus pyogenes* I.S.P. 364-00 but did not show any biological activities against *S. aureus* ATCC 25923, *P. aeruginosa* ATCC 27853, and *E. coli* ATCC 25922 [169]. Interestingly, Slovakian bee pollen extract demonstrated good antibacterial features against a clinical isolate of *E. coli* CCM 3988 [170]. Nonetheless, a general observation in several studies is that the antibacterial action of bee pollen is much higher towards Gram-positive bacteria than their Gram-negative counterparts [169,171,172,173] with some exceptions [174,175]. It is important to note, however, that almost all the antibacterial data were generated in vitro, so it is urgent to confirm the antibacterial efficacy of bee products using currently available vertebrate [176,177,178,179] or invertebrate [180,181,182,183,184,185] in vivo model systems.

In addition to their antibacterial efficacies, bee pollen and bee bread have been reported to display antiviral activities. For example, bee pollen of date palm was found to be active against HSV-1 and HSV-2 [25] and bee pollen extracts of Korean *Papaver rhoeas* was fairly effective against influenza viruses (strains of H1N1, H3N2, and H5N1) [26]. The antiviral activity of bee pollen was most likely due to the action of flavonoids such as luteolin, galangin, kaempferol, and quercetin. Luteolin has been shown as one of the most potent inhibitors of the neuraminidase of influenza virus [26], thus is a prospective anti-influenza drug candidate (as a class of neuraminidase inhibitor). In addition, quercetin was shown to interact with the HA2 subunit of hemagglutinin and inhibit the entry of influenza virus into the host cells [186]. Quercetin-mediated inhibition of hemagglutinin might play a determinant role in the prevention of the hemagglutinin-sialic acid interaction that is required in influenza virus entry. With an increasing rate of viral resistance against the available anti-influenza drugs, such a mechanism shall play a future role in the pharmacological treatment of influenza virus infections.

The emergence of SARS-CoV-2, the causative agent of coronavirus disease (COVID)-19, in late 2019 has increased researchers’ interest in the medical and pharmaceutical potentials of bee products. Several published literatures have encouraged the use of bee products such as honey, propolis, bee pollen, bee bread, and even bee venom, in the management of COVID-19. Lima et al., for example, argued that apitherapy is one of alternative ways that can be tapped to prevent and/or to manage some of the COVID-19-associated symptoms [27]. Indeed, honey and other bee products contain a number of compounds that have been shown effective as antivirals, thus potentially promising against SARS-CoV-2 [27,157,187]. On the basis of such argument, several randomized clinical trials are now carried out to investigate whether the use of honey and propolis in the management COVID-19 are truly effective [27].

## 5. Antiparasitic Potential of Bee Products

Parasitic diseases are still among the most challenging public health issues in the countries with subtropical, tropical, and temperate climates [215,216,217]. One factor contributing to the spread of these infections is the lack of an effective and safe therapy. The current pharmacotherapy options are reported to have significant shortcomings such as being suboptimally active, especially towards the specific form of the parasites, have varying rates of efficacy, have burdensome side effects, need long treatment/administration terms, and the resistance to their action of some parasites [218,219,220]. Considering this scenario, there is a substantial need to find and promote new potent antiparasitic treatments which are affordable and have minimal adverse reactions. 

In recent decades, there has been a keen interest in screening the pharmacological and chemical characteristics of bee-related products, a promising source of natural bioactive substances, as an alternative antiparasitic therapy [221]. Since classical times, bee related products have been popularly used traditionally as herbal remedies for treating some infectious diseases in many communities around the world [222]. In this review, we found that there are four bee-related products i.e., propolis, bee venom, bee pollen and honey that have been extensively studied to uncover their antiparasitic activities against protozoa and worms as the commonest classes of parasites infecting humans. Diverse studies have indicated that bee products are shown to be scientifically effective, via in vitro and/or in vivo tests, in treating a wide variety of infectious diseases such as schistosomiasis, trypanosomiasis (chagas disease), leishmaniasis, toxocariasis, plasmodiasis, toxoplasmosis, blastocystis infection, amebiasis, giardiasis, cryptosporidiosis, and echinococcosis (Table 3). 

The curative properties of bee products have been directly associated to their chemical components. However, the chemical constituents of bee products are complex and differ according to their botanical source and geographical origins as indicated by the regional variations in the antiparasitic activities of the bee products [223,224,225]. Other factors reported to influence the dissimilarity of the physicochemical characteristics of the bee products are the vegetation surrounding the beehive, collection time, soil diversity, geoclimatic conditions or seasons in the collection area, the bee species, and particular flora living at the harvesting location [226,227,228]. Variations in the concentration of effective bee products are also predominantly affected by the type and origins of parasites used in the experiments as well as the preparation method [198,229,230]. There is a wide range of the extraction method applied to obtain, for example, propolis extracts ranging from conventional separation technique using organic solvent such as ethanol to a more sophisticated one such as a supercritical fluid extraction method [231]. The extraction methods can influence the amount of active substances in the extract and therefore, might change the biological activities of the extracts [231]. Lastly, the type of bee products also determines the magnitude of biological properties. Some studies indicated that different varieties of Brazilian propolis such as red, green, and brown have distinct chemical compounds and therefore, have a different potency against parasites parasitizing humans [198,232].

There are several proposed pharmacological mechanism of bee products to act against protozoan infections which are deemed to be facilitated by their flavonoid and phenolic constituents as follows [233,234,235]: (1) Activation of macrophages which kills the parasite via the production of ROS (particularly superoxide dismutase) and nitrogen metabolites [236,237]; (2) The alteration of angiogenesis in the affected tissue [236,238]; (3) Stimulation of immunomodulatory effects, by influencing the production of interferon-γ, tumor necrosis factor α, IL-1, IL-4 and IL-17 [239,240,241]; (4) Induction of apoptosis-like mechanisms in parasites [236]; (5) Membrane disruption in parasites [242].

## 6. Bee Product-Derived Nanoparticles as Potential Therapeutic Agents

Green chemistry principles have recently received much attention for their use in creating biocompatible nanomaterials. Due to the presence of phytoconstituents as stabilizing ligands on their surfaces, nanoparticles prepared by the application of natural product extracts have frequently demonstrated promising bioactivity. Honey bee products such as honey, royal jelly, bee venom, pollen, and beeswax are thought to be promising sources of products to avoid nanoparticle aggregation thus improving the biocompatibility, stability, and biological application. It is possible to functionalize these nanomaterial biomolecules. Metal nanoparticles such as platinum, gold, silver, zinc and others are commonly used nanoparticles in the biomedicine sector. The bactericidal and inhibitory properties of Ag NPs-based nanoparticles against various microbes are quite impressive, along with their high efficiency, strong biocompatibility, easy availability, and low cost which, made them gain significant consideration to scientists and technologists [258]. 

Al-Yousef et al. prepared Ag NPs (AgNPs-G) using bee pollen aqueous extract as a bioreductant during the experiments and found that they demonstrated excellent anti-oxidant properties and worked against different Gram-positive and negative bacteria. They even successfully exerted an anti-proliferative effect against cancer cell lines, including MCF-7 and HepG2 [259]. Magnetite nanoparticles are another type of nanoparticle with antimicrobial properties. According to El-Guendouz et al. magnetite nanoparticles twinned with propolis shows antimicrobial activities against methicillin-resistant strains of *S. aureus* [260]. Honey is another bee product that has antimicrobial, anti-inflammatory, and antioxidant properties. Chen et al. reported a new bioactive component‒vesicle-like nanoparticles (H-VLNs) in honey that shows anti-inflammatory activities [261]. H-VLNs can disrupt a crucial inflammatory signaling platform in the innate immune system by restraining the formation and activation of the nucleotide-binding domain and pyrin domain-containing 3 (NLRP3) inflammasome. In mice, these nanoparticles reduced inflammation and liver damage in an experimentally induced acute liver injury model [261]. 

Like metal nanoparticles, polymeric nanoparticles and liposomes are other types of nanoparticles that are a popular choice as drug delivery vehicles for therapeutic applications in the pharmaceutical area and are safe. A study conducted by Iadnut et al. concluded that ethanolic extract of propolis loaded with polymeric nanoparticles profoundly inhibited the growth of *Candida albicans* [262]. They found that the ethanolic extract of propolis-loaded poly(lactic-co-glycolic acid) nanoparticles can reduce gene-encoding virulence-associated hyphal adhesion proteins of *C. albicans*, which further attenuates the fungal virulence [262]. In another study, do Nascimento et al. investigated the immunosuppressive activity of “multiple-constituent extract in the co-delivery system” against leishmaniasis by loading using Brazilian red propolis extract into polymeric nanoparticles [263]. Various dosage forms of red propolis extract loaded with nanoparticles were tested and discovered to be a potential intermediate product for the preparation of various drugs for diseases like leishmaniasis. 

Bee venom is gaining popularity for its antipathogenic, anticancer, anti-tumor activities. Alalawy et al. prepared fungal chitosan nanoparticles loaded with bee venom and demonstrated that such bee-venom nanoparticle preparation was significantly potent as a natural anti-proliferative agent against cervical cancer [28]. In addition to that, Saber et al. used bee venom loaded with chitosan to successfully treat amoebiasis in mice [264], indicating that, bee venom possesses antiparasitic properties in addition to its anticancer properties.

## 7. Concluding Remarks and Future Directions

Bee products such as honey, propolis, bee pollen, royal jelly, beebread, beeswax, and bee venom have been broadly used in traditional healing practices. With their potential medical and pharmaceutical properties, increasing interest in bee products has been seen in the last century. With the advancements in research tools and our great progress in the understanding of biological processes, the main active component(s) responsible for the anticancer, antibacterial, antiviral as well as antiparasitic properties of bee products need to be clearly elucidated in a standardized way in order to improve the application of bee products in disease management. The issue of standardization has also been hampering the use of bee products not only in pharmaceuticals but also in cosmetics and food industries. Furthermore, there is also a need to determine the optimal dose of bee products and how to use the products to treat cancer as well as infections. This information is substantial in order to bridge the experimental results from the bench to the bedside.

## Figures and Tables

**Table 1:** Anticancer potentials of selected bee products.

Bee Products	Identified Substance(s)	Tested Cell Lines	Type(s) of Cancer	Possible Mechanism(s)	Ref.
Honey	Gallic acid, caffeic acid, syringe acid, chlorogenic acid, *p*-coumaric acid, ferulic acid, catechin, quercetin, chrysin	PC-3	Human prostate adenocarcinoma	Not examined	[106]
Honey (Tualang)	Not examined	MCF-7, MDA-MB-231	Breast cancer	Modulation of apoptotic signalling pathway by enhancing the expression of p53, p21 and FADD protein	[112]
Honey (Manuka)	Not examined	MCF-7, MDA-MB-231	Breast cancer	Reduction of interleukin (IL)-6 and inhibition of pY-STAT3 signalling. Inhibition of cell invasion and migration	[102]
Honey (Chesnut honey)	3–2′-pyrrilonidinyl-kynurenic acid	CRPC	Castration-resistant prostate cancer	Induction of apoptosis via caspase-3	[111]
Bee pollen	<65 kDa peptides	ChaGo-K-1	Human bronchogenic carcinoma	Induction of apoptosis (biomolecular pathway not known yet)	[121]
Propolis	Pinocembrin as the major constituent	MCF-7, HCT and THP-1	Breast cancer, human colon cancer, and human leukemia model	Enhanced production of interleukin-10 (IL-10) and decreased production of TNF-a and IL-6	[117]
Propolis	Triterpenes, steroids derivatives, and diterpenes	Jurkat HEP-62 SW-756	T-lymphocyte leukemia, human liver carcinoma, Squamose carcinoma.	Interaction with microtubules; induction of tubulin depolymerisation	[114]
Propolis	Chrysin, galangin and *p*-coumaric acid	CAL-27	Human tongue squamosa cancer	Activation of apoptotic cascades via caspase-3, -8 and -9	[116]
Propolis	Chrysin, caffeic acid, *p*-coumaric acid and ferulic acid	CAL-27	Human tongue squamosa cancer	Decreased level of proline in cancer cells via proline dehydrogenase/proline oxidase activity	[118]
Propolis	3-O-methylquercetin, chrysin, caffeic acid, CAPE, galangin and pinocembrin	MCF-7, HGC-27, A549	Breast cancer, human gastric carcinoma, human lung adenocarcinoma	Induction of apoptosis, promotion of cell cycle arrest via activation of p21	[140]
Royal jelly	10-hydroxy-2-decenoic acid	MCF-7	Breast cancer	Not examined	[133]
Royal jelly	Not examined	Mouse 4T1	Mouse mammary carcinoma	Increased concentration of IL-2 and interferon (INF)-α; decreased level of IL-10	[132]
Royal jelly	10-hydroxy-2-decenoic acid	U-937	Leukemia	Induce secretion of cytokines by mononuclear cells	[138]
Bee bread	Polyunsaturated fatty acids (51%) and monounsaturated fatty acids (9.9%)	Caco-2 PC-3	Human colorectal adenocarcinoma, Human prostate adenocarcinoma	Not examined	[139]
Bee bread	Flavonoids and polyphenols	MCF-7 HeLa	Breast cancer Cervical cancer	Not examined	[89]
Bee venom	Mellitin	AGS	Gastric cancer	Disruption of cell membrane causing necrosis to the affected cells	[127]
Bee venom	Mellitin	A375	Human lung cancer	Induction of apoptosis via activation of caspase-9 and caspase-3, inhibition of invasion and migration of melanoma cells through interference of f-actin reorganisation and epidermal growth factor receptor (EGFR) activity	[124]

**Table 2:** Antibacterial activity of selected bee products.

Bee Products	Country	Bacteria	Assay Method	Results	Ref.
*Leptospermum* honey (80 different honeys derived from *Leptospermum* species)	Australia	*Staphylococcus aureus* (ATCC 25923)	In vitro (phenol equivalence assay)	Majority of Australian *Lectospermum* honey tested in the experiments demonstrated non-peroxide antibacterial activity (NPA) and to a greater extent correlates to their high content of methylglyoxal (MGO) and dihydroxyacetone (DHA)	[188]
21 types of honey of different botanical source, derived from the Olympus mountain	Greece	Clinical isolates of methicillin-resistant *S. aureus* (strain 1552) and carbapenem-resistant *P. aeruginosa* (strain 1773)	In vitro (agar well diffusion assay and microtiter plate assay)	All honey samples yielded antibacterial activity against both tested bacteria. Some honey samples were active in a manner dependent on the presence of hydrogen peroxide and proteinaceous compounds	[189]
*Sesamum indicum* honey (seven types of sesame honey obtained from different location in West Bengal)	India	*Salmonella enterica* serovar Typhi, *S. enterica* serovar *Typhimurium*, *Escherichia coli* and *Vibrio cholerae*	In vitro (disc diffusion assay and microbroth dilution assay)	The antibacterial activity of sesame honey against the tested enteropathogens was good, with the best recorded activity was against both Salmonella species	[190]
New Zealand’s Manuka Honey (UMF +20, UMF +16, +10), Sidr honey, and *Nigella sativa* honey	New Zealand, Saudi Arabia	Methicillin sensitive- (ATCC 29213 and 10 strains of MSSA clinical isolates) and methicillin resistant *S. aureus* (ATCC 26112 and 10 strains of MRSA clinical isolates)	In vitro (disc diffusion assay and microbroth dilution assay)	Manuka honey demonstrated bactericidal activity against both MSSA and MRSA while Sidr and *Nigella sativa* honey yielded only bacteriostatic effect at tested concentrations	[191]
*Melipona beecheii* honey (Cuba); Manuka honey (New Zealand), *Apis mellifera* honey (Cuba), and African *A. mellifera* honey (Kenya)	New Zealand, Cuba, and Kenya	51 clinical isolates (34 Gram-positive, 17 Gram-negative)	In vitro (microtiter plate assay for antibiofilm activity)	All honey tested in the study demonstrated good antibacterial and antibiofilm activity with Cuban *M. beecheii* honey had the highest activity in both	[192]
Ten samples of honey of different origins (polyfloral, linden, acacia, manna, and sunflower)	Romania	*S. aureus, Staphylococcus epidermidis*, *S. enterica* serovar Typhimurium, *Bacillus cereus*, *B. subtilis, Pseudomonas aeruginosa*, *E. coli*, and *Listeria monocytogenes*	In vitro (disc diffusion assay)	All honey samples demonstrated good antibacterial activity against all tested pathogens with *S. aureus* and *P. aeruginosa* were the most sensitive ones. It seems that the origins and the color of honey, but not acidity, play a role in the antibacterial activity of honey	[193]
*Apis mellifera* ligustica propolis (extracted using methanol)	Australia	*S. aureus* (ATCC 25923) and *Klebsiella pneumoniae* (ATCC 13883)	In vitro (agar diffusion and broth dilution assays)	The examined Australian propolis demonstrated antibacterial effect against *S. aureus* (bactericidal) but did not yield any effect on the *K. pneumoniae*	[194]
Brazilian brown and green propolis (extracted either using ethanol, hexane, or dicholometane)	Brazil	*S. aureus* (ATCC 6538), *B. subtilis* (ATCC 6633) and *Micrococcus luteus* (ATCC 10240)	In vitro (micro-dilution assay)	Of all samples examined in the study, the dichloromethane extract of both brown propolis and green propolis yielded the highest antibacterial effect against the tested pathogens	[195]
Propolis of *T. fiebrigi* bees (extracted using ethanol)	Brazil	*S. aureus* (ATCC 43300), *S. aureus* (ESA 654), *S. epidermidis* (ATCC 12228), *S. epidermidis* (ESA 675), *Enterococcus faecalis* (ATCC 43300), *E. faecalis* (ESA 553), *K. pneumonia* (ATCC 4352), *K. pneumoniae* (ESA 154), *P. aeruginosa* (ATCC 15442), *P. aeruginosa* (ESA 22), *Proteus mirabilis* (ATCC 43300) and *P. mirabilis* (ESA 37)	In vitro (micro-dilution assay)	The Brazilian propolis demonstrated antibacterial effect against all tested microorganisms but mainly active against Gram-positive bacteria	[196]
Red propolis of Africanized *Apis mellifera*	Brazil	Standard strains of *E. coli* (ATCC 25922) and *S. aureus* ATCC 6538 and the clinical isolates of *E. coli* 06 (EC06), *P. aeruginosa* 03 (PA03), *P. aeruginosa* 24 (PA24) and *S. aureus* 10 (SA10)	In vitro (micro-dilution assay)	The Brazilian red propolis demonstrated antibacterial activity against all tested pathogens, including the clinical isolate ones. The range of MIC values was dependent on the pathogen species (128–512 μg/mL for *E. coli* strains), (64 μg/mL to ≥1024 μg/mL for *S. aureus* strains), and (512 μg/mL for *P. aeruginosa* strains)	[197]
Red propolis, green propolis, and brown propolis (traditionally extracted using ethanol or supercritical extraction method)	Brazil	*S. aureus* (ATCC 25923) and *Enterococcus* sp. (ATCC 29712), *Klebsiella* sp. (ATCC 1706/700603), and *E. coli* (ATCC 25922)	In vitro (micro-dilution assay)	Of all samples, Brazilian red propolis extract yielded the highest antibacterial activity. Green propolis extract demonstrated weak to moderate antibacterial activity for most samples and brown propolis extract did not yield any antibacterial effect against the tested strains. None of the examined samples was active against *E. coli*	[198]
19 propolis samples (collected from six different regions and extracted using methanol)	Chile	Methicillin-sensitive *S. aureus* (ATCC 25923), methicillin-resistant *S. aureus* (ATCC 43300), *E. coli* (ATCC 25922), and the clinical isolates of *Pseudomonas* sp., *E. coli*, *P. mirabilis*, *Salmonella enteritidis*, *Salmonella* sp. and *Yersinia enterocolítica*	In vitro (micro-dilution assay)	The antibacterial effect of propolis obtained from central valley was better than the ones collected from the Andreas slopes or the coastal areas. The samples’ MICs were ranging from 31.5 to >1000 µg/mL and the ones with MIC ≤ 62.5 µg/mL demonstrated good antibacterial effect against *Pseudomonas* sp., *E. coli*, *S. enteritidis*, and *Y. enterocolitica*	[199]
Propolis collected from different geographical regions (extracted using either ethanol or water)	Germany, Irlandia, Czech Republic	*S. aureus*, *Staphylococcus saprophyticus*, *S. epidermidis*, *S. pneumoniae*, *Streptococcus pyogenes*, *Streptococcus oralis*, *Streptococcus agalactia*, *Streptococcus thermophiles*, *B. subtilis*, *Enterococcus casseliflavus*, *K. pneumonia*, *Klebsiella oxytoca*, *E. coli*, *E. coli* O157.H7, *P. aeruginosa*, *Salmonella choleraesuis*, *Shigella flexneri*, *Haemophilue influenza*, *Acinetobacter baumanii*, *Burkholderia cepacia*, *Y. enterocolitis*, *Enterobacter cloacae*, one strain of MRSA, and one strain of Vancomycin-resistant enterococci (VRE)	In vitro (micro-dilution assay)	Both ethanol and water propolis extracts demonstrated good antimicrobial activity against most of Gram-positive bacteria (range of MICs: 0.08–5 mg/mL), with the Irish propolis yielded the highest bactericidal effect followed by Czech and German. All propolis extracts demonstrated moderate antibacterial against MRSA and VRE and also against β-lactamase positive *H. influenzae*, and *S. pneumoniae*. Propolis ethanol extract, but not water extract, yielded moderate antibacterial activity against Gtam-negative pathogens tested in the study (MICs: 0.6–5 mg/mL)	[200]
Propolis and bud poplar resins (extracted using ethanol)	Italy	*P. aeruginosa* PAO1 (ATCC 15692) and transgenic *P. aeruginosa* (P1242) with the luciferase gene and luciferin substrate (under the control of a constitutive P1 integron promoter)	In vitro (micro-dilution assay)	Both ethanol extracts (propolis and bud poplar resins) demonstrated good antibacterial activity against *P. aeruginosa* biofilm and negatively affected the swimming and swarming motility properties of *P. aeruginosa*	[201]
24 propolis samples (collected from different geographical location in Morocco; hydro-alcoholic extracts)	Morocco	*S. aureus* (ATCC 6538) and three clinical isolates of MRSA (MRSA2, MRSA 15, and MRSA 16)	In vitro (disk diffusion method)	Propolis extract (MIC 0.36 mg/mL) was able to attenuate the virulence of *S. aureus* ATCC 6538 and the MRSA strains. The impairment of biofilm formation was also observed	[202]
Propolis of *Populus alba, P. nigra*, *P. tremula*, *Acer pseudoplatanus*, *Betula verucosa*, *Pinus silvestris*, and *Aesculus hippocastanum* (extracted using methanol or dichlorometane)	Poland	*S. aureus* (ATCC 25923), *S. epidermidis* (ATCC 12228), *P. aeruginosa* (ATCC 227853), *E. coli* (ATCC 25922), *E. cloacae* (ATCC 13047), and *K. pneumoniae* (ATCC 13883)	In vitro (disk diffusion method)	The dichloromethane extract of propolis demonstrated good antibacterial activity against all tested pathogens (MICs: 0.90–1.34 mg/mL)	[203]
Green propolis (extracted using ethanol, methanol, diethyl ether or water)	Taiwan	*S. aureus* (BCRC 10780, BCRC 10781 and BCRC 10451), MRSA (ATCC 43300), *B. subtilis* (BCRC 10255), *L. monocytogenes* (BCRC 14845), *P. aeruginosa* (BCRC 10944), and *E. coli* (BCRC 10675)	In vitro (micro-dilution assay)	Taiwanese green propolis extracts demonstrated good antibacterial activity against MRSA and all propolins, particularly propolin C, yielded good efficacy against *S. aureus*, *B. subtilis*, and *L. monocytogenes*	[204]
29 bee pollen samples (collected during the dry seasons of 2016, 2017, and 2018; extracted using ethanol)	Chile	*S. aureus* (ATCC 25923), *P. aeruginosa* (ATCC 27853), *E. coli* (ATCC 25922), and *S. pyogenes* (I.S.P. 364-00)	In vitro (disk diffusion method and broth dilution method)	All bee pollen extracts (collected in three different years) demonstrated good antibacterial activity against *S. pyogenes* but did not yield antibacterial effect on *S. aureus*, *P. aeruginosa*, and *E. coli*	[169]
Three Greek bee pollen (collected from from *Cistus creticus* L. (rock rose) at three different locations; extracted using ethanol, butanol, dichloromethane, or cyclohexane)	Greece	*S. aureus* (ATCC 25923), *S. epidermidis* (ATCC 12228), *P. aeruginosa* (ATCC 227853), *E. coli* (ATCC 25922), *E. cloacae* (ATCC 13047), and *K. pneumoniae* (ATCC 13883)	In vitro (micro-dilution assay)	The butanol extract demonstrated good antibacterial activity against all pathogens tested in the study, probably due to the high content of flavonoids, such as quercetin and kaempferol glucosides. No antimicrobial activity was seen in both cyclohexane and dichlorometane extracts	[205]
Bee bread and propolis of Stingless bee (*Heterotrigona itama* species; extracted using ethanol or hexane)	Malaysia	*S. aureus, B. subtilis, E. coli*, and *Salmonella*	In vitro (disk diffusion method and broth dilution method)	All bee bread and propolis extracts demonstrated good antimicrobial activity against all tested bacteria (MIC: <6.67–33.33 µL/mL), with higher preference to Gram-positive (*S. aureus* and *B. cereus*) than Gram-negative bacteria (*E. coli* and *Salmonella*). Ethanol extracts yielded stronger antibacterial activity than their hexanic counterparts	[206]
*Castanea sativa* Mill. pollen grains (collected at Erfelek (4 sites) and Ayancık (5 sites) district of Sinop; extracted using methanol)	Turkey	S. *aureus* (ATCC 6538), MRSA, *E. faecalis* (ATCC 51299), *M. luteus*, *Bacillus cereus* (7064), Vancomycin-resistant *Enterococcus* (VRE), *E. coli* (ATCC 11293), and *K. pneumonia*	In vitro (disk diffusion method)	Bee pollen extracts yielded higher antibacterial activity against Gram-positive bacteria than their Gram-negative counterparts	[207]
Royal jelly sample	Singapore	*Fusobacterium mucleatum*, *Aggregatibacter actinomycetemcomitans*, *Porphyromonas gingivalis*, and *Prevotella intermedia*	In vitro (micro-dilution assay)	Royal jelly demonstrated good antibacterial activity against periodontopathic bacteria tested in the study	[208]
Royal jelly sample (Yamada Bee Farm, Okayama, Japan)	Japan	*P. aeruginosa* (PAO1) wild-type strain and four clinical isolates (TUH-54, TUH-124, TUH-188, and TUH-213)	In vitro (micro-dilution assay and microtiter plate biofilm assay)	Royal jelly did not yield antipseudomonal activity at concentration of 50% *w*/*v*. However, at concentration of less than 25%, antiadherent activity was observed on both the abiotic surface and the epithelial cell model	[209]
Bee bread sample (hydromethanolic extract)	Morocco	*S. aureus* (ATCC 6538), *B. cereus* (food isolate), *L. monocytogenes* (NCTC 7973), *E. coli* (ATCC 35210), *E. cloacae* (human isolate), *S.* Typhimurium (ATCC 13311)	In vitro (micro-dilution assay)	Hydromethanolic extract of bee bread demonstrated antibacterial activity against all tested pathogens (MIC: 0.04–0.175 mg/mL; MBC: 0.08–0.35 mg/mL)	[210]
Bee bread (five different regions of Ukraine; extracted using ethanol)	Ukraine	*S. aureus* (CCM 4223), *E. coli* (CCM 3988), *S. enterica* subs. *enterica* (CCM 3807), and *Bacillus thuringiensis* (CCM 19)	In vitro (disk diffusion method)	All bee bread samples yielded good antibacterial activity against antibiotic-resistant Gram-positive and Gram-negative bacteria tested in the study. The antibacterial strength of bee bread samples on certain bacteria was varied, mainly depends on the geographical location of sample collection	[211]
*Apis mellifera* venom (compared to mellitin)	n/a	*Borrelia burgdorferi* strain B31 (ATCC 35210)	SYBR Green I/Propidium Iodide assay and biofilm assay	Both bee venom and mellitin demonstrated good antimicrobial activity against the free form of *B. burgdorferi* and the biofilm-associated form	[162]
*Apis mellifera* venom (compared to mellitin)	Brazil	*S. aureus* (ATCC 13565, ATCC 14558, ATCC 19095, and ATCC 23235, all Enterotoxigenic), and five clinical isolates of MRSA (recovered from human specimens)	The resazurin microtiter assay (REMA)	Apitoxin and melittin yielded antibacterial activity against MRSA (MIC: 7.2 μg/mL, and 6.7 μg/mL, respectively). Apitoxin and melittin had no effect on the production of enterotoxin and/or its release.	[212]
*Apis mellifera* venom (commercial, freeze-dried)	n/a	*Pseudomonas putida* (ATCC 700008), *Pseudomonas fuorescens* (NCIMB 9046) and *E. coli* K-12, MG1655 (ATCC 47076),	In vitro viability assay and biochemical analysis	Bee venom exerted its antibacterial activities against the tested pathogens via alteration in the membrane permeability, destruction of bacterial cell wall, cell contents leakage, and inactivation of bacterial metabolic activity leading to cell death	[213]
*Apis mellifera* venom (apitoxin)	Ecuador	*Salmonella* (CECT 4395) and *L. monocytogenes* (CECT 934) and 49 *S. enterica* and 7 L. monocytogenes strains isolated from poultry products	In vitro (micro-dilution assay)	Apitoxin is effective against foodborne pathogens tested in the study (MIC range: 16–32 µg/mL for *L. monocytogenes* and 256–1024 µg/mL for *S. enterica*)	[214]

**Table 3:** Antiparasitic effect of selected bee products.

Bee Products	Country	Parasites	Assay Method	Results	Refs.
Propolis extracts (12 samples from different location in Libya)	Libya	*Trypanosoma brucei* (s427); *Leishmania donovani*; *Plasmodium falciparum*	In vitro	All the extracts are to some extent effective against all of the tested protozoa. There are regional variations in the antiparasitic properties	[223]
Ethanolic extracts of European propolis (35 different samples)	Europe	*Trypanosoma brucei 427WT*; *Trypanosoma brucei* B48 (resistant strain); *Trypanosoma congolense*; *Leishmania mexicana WT*; *Leishmania mexicana* C12Rx (resistant strain)	In vitro	All extracts display high level activities against the parasites. Yet, there are regional variations in the antiparasitic properties	[225]
Hydroethanolic red propolis extracts	Brazil	*Trypanosoma cruzi*; *Leishmania braziliensis vianna*; *Leishmania infantum promastigotes*	In vitro	The extracts exhibit strong cytotoxic properties against the protozoan parasites	[227]
Ethanolic extracts of red propolis	Brazil	*Schistosoma mansoni*	In vitro, in vivo	Propolis displays antischistosomal properties by decreasing motility and producing: mortality of adult parasites; morphological disruptions in the schistosomes’ tegument; and substantial impairment in egg generation. Propolis also significantly lower the worm and egg burden in both early and chronic *S. mansoni* murine infection model	[229]
Ethanolic extracts of red propolis	Brazil	*Trypanosoma cruzi*	In vitro	Ethanolic propolis extracts have high inhibitory activity against *T. cruzi*	[198]
Dry, alcoholic, and glycolic green propolis extracts	Brazil	*Leishmania* (*Viannia*) *braziliensis promastigotes* and *amastigotes*	In vitro	The extracts exhibits antileishmanial activity against promastigotes and amastigotes stages of the parasite	[237]
Ethanolic extracts of red and green propolis	Brazil	*Leishmania (Vianna) braziliensis*	n.a	Both propolis extracts exhibits leishmanicidal effect in a dose-dependent manner. Yet, the red propolis extract displays a higher efficacy than the green extract	[231]
Hydroethanolic extract of red propolis	Brazil	*Leishmania chagasi* promastigote; *Leishmania amazonensis* promastigote	In vitro	The extract exhibits leishmanicidal potency againts the parasites	[243]
Hydroalcoholic extract of Brazilian propolis	Brazil	*Leishmania (Viannia) braziliensis*	In vitro	Propolis extract shows immunomodulatory effects, by enhancing IL-4 and IL-17 and lowering IL-10, and therefore, may protect against Leishmania infection and clear the parasite	[239]
Hydroalcoholic extract of Brazilian green propolis	Brazil	*Leishmania amazonensis* promastigotes and amastigotes	In vitro In vivo	Propolis extract decreases the viability of *L. amazonensis* amastigotes and promastigotes. The extract also decreases the parasites and stimulates the macrophage recruitment into the lesion caused by the *L. amazonensis*	[236]
Degradation products of major compounds of green propolis: Z-artepillin C and Z-*p-*coumaric Acid	Brazil	*Leishmania amazonensis* promastigotes and amastigotes	In vitro	Both Z-artepillin C and Z-*p*-coumaric acid display a promising and significant activity against *L. amazonensis*	[244]
Hydroalcoholic extract of *Melipona fasciculata* geopropolis	Brazil	*Leishmania amazonensis* promastigotes and amastigotes	In vitro	Geopropolis has an antileishmanial effect and effective in reducing the number of the *L. amazonensis* promastigotes and amastigotes	[233]
Isolated compound of Bee pollen monofloral	Brazil	*Leishmania amazonensis*	In vitro	The isolated compound identified as the biflavonoid rhusflavone shows high antileishmanial effect against *L. amazonensis* promastigotes and amastigotes	[234]
Bee venom and propolis	Egypt	*Schistosoma mansoni*	In vivo	Bee venom and propolis exerts anti-schistosomal activities by substantially lowering the mean total numbers of worm, mean values of immature and mature egg as well as the ova count in hepatic tissue	[245]
Egyptian propolis ethanolic extract	Egypt	*Toxocara vitulorum*	In vitro	The extract exhibits anthelmintic efficacy and the nematicidal effect is dose-dependent	[246]
Egyptian propolis ethanolic extract	Egypt	*Blastocystis* spp.	In vitro	The extract presents a notable obstructive effect on the growth of *Blastocystis* parasites	[247]
Egyptian propolis ethanolic and water extract	Egypt	*Cryptosporidium* spp.	In vivo	The prophylactic and therapeutic administration of the extracts moderately effective in reducing the oocysts shedding on cryptosporidiosis infected rats	[248]
Egyptian propolis ethanolic extract	Egypt	*Toxoplasma gondii*	In vivo	Propolis markedly decreases the amount of IL-1β, IL-6, and TNFα in *T. gondii* infected models	[249]
Egyptian propolis ethanolic extract	Egypt	*Giardia lamblia*	In vivo	Propolis markedly reduce the *G. lamblia* trophozoites count	[250]
*Ziziphus spina-christi* honey; *Acacia nilotca* honey; *Acacia seyal* honey; *Cucurbita maxima* honey	Saudi Arabia	*Entamoeba histolytica*; *Giardia lamblia*	In vitro	All honeys are potentially effective to be used as antiamoebic and antigiardial agents since they can halt the growth of the trophozoites	[47]
Ethanolic extraction of Saudi propolis	Saudi Arabia	*Trypanosoma brucei*	In vitro	The extract indicates a significant anti-trypanosomal activity	[251]
*Capparis spinosa* honey	Saudi Arabia	*Toxoplasma gondii*	In vivo	Honey elevates the amount of antibody titer and the cytokines (IFN-γ, IL-1, and IL-6) in *T. gondii* infected rats	[241]
Ethanol and dichloromethane Propolis extracts	Iran	*Plasmodium falciparum*	In vitro	All extracts show concentration-dependent anti-plasmodial activity. Dichloromethane extract has the most potent inhibitory effect	[252]
Ethanolic extract of propolis	Iran	*Leishmania major*	In vitro, In vivo	Both tests indicate that the extract has an effective antileishmanial activity against *L. major.* The extract reduces the number of promastigotes and decreases the size of ulcers significantly	[253]
Ethanolic extract of algerian propolis	Algeria	*Echinococcus granulosus*	In vitro, In vivo	Both tests indicate that the extract is an effective antihydatic scolicidal effect since it has a major scolicidal activity against *E. granulosus* at all tested concentration and reduces cystic echinococcosis development in in vivo model	[254]
Methanolic extracts of propolis (ten different propolis from different geographical area in Bolivia)	Bolivia	*Leishmania amazonensis*; *Leishmania braziliensis*	In vitro	All propolis extracts show growth inhibition against both protozoa. Propolis with rich phenolic contents shows the best antiprotozoal effect	[255]
Methanolic extracts of propolis (three different propolis from different geographical area in Ecuador)	Ecuador	*Leishmania amazonensis*	In vitro	All propolis extracts show growth inhibition against the protozoa. Propolis with the richest flavonoids contents shows the best antiprotozoal effect	[235]
Ethanolic extracts of propolis (twelve different propolis from eight different geographical area in Nigeria)	Nigeria	*Trypanosoma brucei* (s427, wild-type); *Trypanosoma brucei* (B48, resistant strain); *Trypanosoma brucei* (aqp2/aqp3 null, resistant strain)	In vitro	The extracts are active against all the tested parasites	[224]
Isolated phenolic compounds of Nigerian red propolis	Nigeria	*Trypanosoma brucei* (s427, wild-type); *Trypanosoma brucei* (B48, resistant strain); *Trypanosoma brucei* (aqp2/aqp3 null, resistant strain)	In vitro	The extract displays moderate to high antitrypanosomal effectivity against all the tested parasites	[256]
Tanzanian propolis ethanolic extract	Tanzania	*Trypanosoma brucei* (s427, wild-type); *Trypanosoma brucei* (B48, resistant strain)	In vitro	The extract displays antitrypanosomal potency against both parasites	[257]
Zambian propolis ethanolic extract	Zambia	*Trypanosoma brucei* (s427, wild-type); *Trypanosoma brucei* (B48, resistant strain)	In vitro	The extract displays antitrypanosomal activity against the wild type of *T. brucei* and the multi-drug resistant clone	[257]

## Data Availability

Available data are presented in the manuscript.
